# The pan-HER family tyrosine kinase inhibitor afatinib overcomes HER3 ligand heregulin-mediated resistance to EGFR inhibitors in non-small cell lung cancer

**DOI:** 10.18632/oncotarget.5286

**Published:** 2015-09-15

**Authors:** Kimio Yonesaka, Keita Kudo, Satomi Nishida, Takayuki Takahama, Tsutomu Iwasa, Takeshi Yoshida, Kaoru Tanaka, Masayuki Takeda, Hiroyasu Kaneda, Isamu Okamoto, Kazuto Nishio, Kazuhiko Nakagawa

**Affiliations:** ^1^ Department of Medical Oncology, Kinki University School of Medicine, Osaka, Japan; ^2^ Center for Clinical and Translational Research, Kyushu University, Fukuoka, Kyushu, Japan; ^3^ Department of Genome Biology, Kinki University School of Medicine, Osaka, Japan

**Keywords:** afatinib, erlotinib, heregulin, non-small cell lung cancer, human epidermal growth factor receptor

## Abstract

Afatinib is a second generation epidermal growth factor receptor-tyrosine kinase inhibitor (EGFR-TKI) characterized as an irreversible pan-human EGFR (HER) family inhibitor. Afatinib remains effective for a subpopulation of patients with non-small cell lung cancer (NSCLC) with acquired resistance to first generation EGFF-TKIs such as erlotinib. Heregulin activates HER3 in an autocrine fashion and causes erlotinib resistance in NSCLC. Here we examine whether afatinib is effective against heregulin-overexpressing NSCLCs harboring EGFR activating mutations. Afatinib but not erlotinib decreased EGFR mutant NSCLC PC9HRG cell proliferation *in vitro* and in mouse xenografts. Afatinib inhibited phosphorylation of the cell signaling pathway proteins HER3, EGFR, HER2, and HER4, likely by prevention of trans-phosphorylation as HER3 kinase activity is inadequate for auto-phosphorylation. Afatinib, unlike erlotinib, inhibited AKT activation, resulting in elevated apoptosis in PC9HRG cells. Clinically, a subpopulation of 33 patients with EGFR mutations and NSCLC who had received first generation EGFR-TKIs exhibited elevated plasma heregulin levels compared to healthy volunteers; one of these achieved a response with afatinib therapy despite having previously developed erlotinib resistance. Afatinib can overcome heregulin-mediated resistance to erlotinib in EGFR mutant NSCLC. Further studies are necessary to determine whether heregulin can predict afatinib efficacy after development offirst generation EGFR-TKI resistance.

## INTRODUCTION

Epidermal growth factor receptor (EGFR) is a molecular target of oncotherapy in certain kinds of cancer including non-small cell lung cancer (NSCLC) [1]. EGFR inhibitors are classified into tyrosine kinase inhibitors or monoclonal anti-EGFR antibodies [2]. EGFR tyrosine kinase inhibitors (EGFR-TKI) such as gefitinib or erlotinib show drastic efficacy in patients with NSCLC harboring EGFR activating mutations [3, 4]. The constructive alteration of EGFR resulting from genomic mutation causes preferential ATP binding with its kinase domain and spontaneous EGFR activation. However, tumors do not always respond to EGFR-TKI therapy despite the presence of EGFR activating mutations. Furthermore, all patients ultimately become refractory to EGFR-TKI treatment after their initial response. Previous research has elucidated several underlying mechanisms in the resistance to EGFR inhibitors [5]. For example, approximately 50% of patients with NSCLC harboring EGFR activating mutations were shown to have developed a T790M substitution EGFR secondary mutation upon their acquisition of resistance to EGFR-TKIs [6]. In addition, MET amplification, human EGFR 2 (HER2) amplification, or hepatocyte growth factor overexpression have also been observed to cause EGFR-TKI resistance in NSCLC [7–9]. Therefore, current clinical investigations seek to identify a novel treatment strategy for conquering EGFR-TKI resistance.

Afatinib is a second generation EGFR-TKI, which can irreversibly bind to EGFR tyrosine kinase and more potently inhibit its activation than can first generation EGFR-TKIs [10]. In phase III clinical trials or combined analyses, afatinib was shown to significantly prolong progression-free survival as well as overall survival compared to standardized cytotoxic chemotherapy in patients with advanced NSCLC harboring EGFR-activating mutations, especially exon 19 deletions [11–13]. Pre-clinical studies have suggested that second generation EGFR-TKIs are effective in NSCLC cells harboring EGFR exon 20 T790M mutations, which causes resistance to first generation EGFR-TKIs [10, 14]. Dacomitinib represents one such second generation EGFR-TKI. However, dacomitinib resistant cell line models were shown to have developed focal amplification of EGFR that preferentially involved the T790M-containing allele [15]. Furthermore, in clinical studies, afatinib exhibited only a limited efficacy in patients with EGFR mutant NSCLC who acquired a resistance to first generation EGFR-TKIs. Asapproximately half of these are likely to possess an EGFR T790M mutation [16, 17], these results suggest that afatinib was not sufficient to overcome T790M-induced resistance to EGFR-TKIs. Additionally, afatinib has more and greater toxicities including diarrhea or rash compared to those associated with first generation EGFR-TKIs because of its irreversible inhibition of wild-type EGFRs in normal cells [17]. In contrast, third generation EGFR-TKIs such as AZD9291 and CO-1686 were shown to exhibit enhanced efficacy against NSCLCs with EGFR T790M mutations and less overall toxicity as a result of wild-type EGFR inhibition being unaffected by these compounds [18, 19].

However, a unique characteristic of afatinib is its ability to inhibit the tyrosine kinase activity of members of the pan-HER family including HER1 (also known as EGFR), HER2, and HER4. In this regard, afatinib is potentially more effective compared to first or third generation EGFR-TKIs, which inhibit only the EGFR tyrosine kinase.

Heregulin is a ligand for HER3 and is aberrantly overexpressed in certain cancer cells including those from NSCLC, in which HER3 is activated in an autocrine fashion [20–22]. Previously, both preclinical and clinical studies have suggested that heregulin could cause resistance to EGFR inhibitors in NSCLC and colorectal cancer (CRC) [20, 22–26]. Heregulin expression levels varies among those cancers, and patients with higher heregulin-expressing cancer cells were shown to exhibit more refractory and shorter progression free-survival with EGFR-TKIs such as erlotinib in NSCLC and with anti-EGFR antibodies such as cetuximab in CRC compared to patients with lower heregulin expression [23, 25, 26].

In contrast to first generation EGFR-TKIs, the efficacy of afatinib against NSCLCs with EGFR-TKI resistance because of heregulin-overexpression has never been evaluated. In this study, we aimed to examine whether afatinib could efficiently prevent cell proliferation in heregulin-overexpressing NSCLC cells harboring EGFR-activating mutations. In addition, we analyzed cell-signaling pathway activation in order to elucidate the mechanism by which afatinib might overcome EGFR-TKI resistance in heregulin-overexpressing NSCLCs. Finally, we examined the clinical efficacy of afatinib in patients with NSCLC and EGFR mutations in correlation with their level of heregulin expression.

## RESULTS

### Heregulin-overexpressing PC9HRG cells were resistant to erlotinib, but maintained afatinib sensitivity

**Figure 1:**
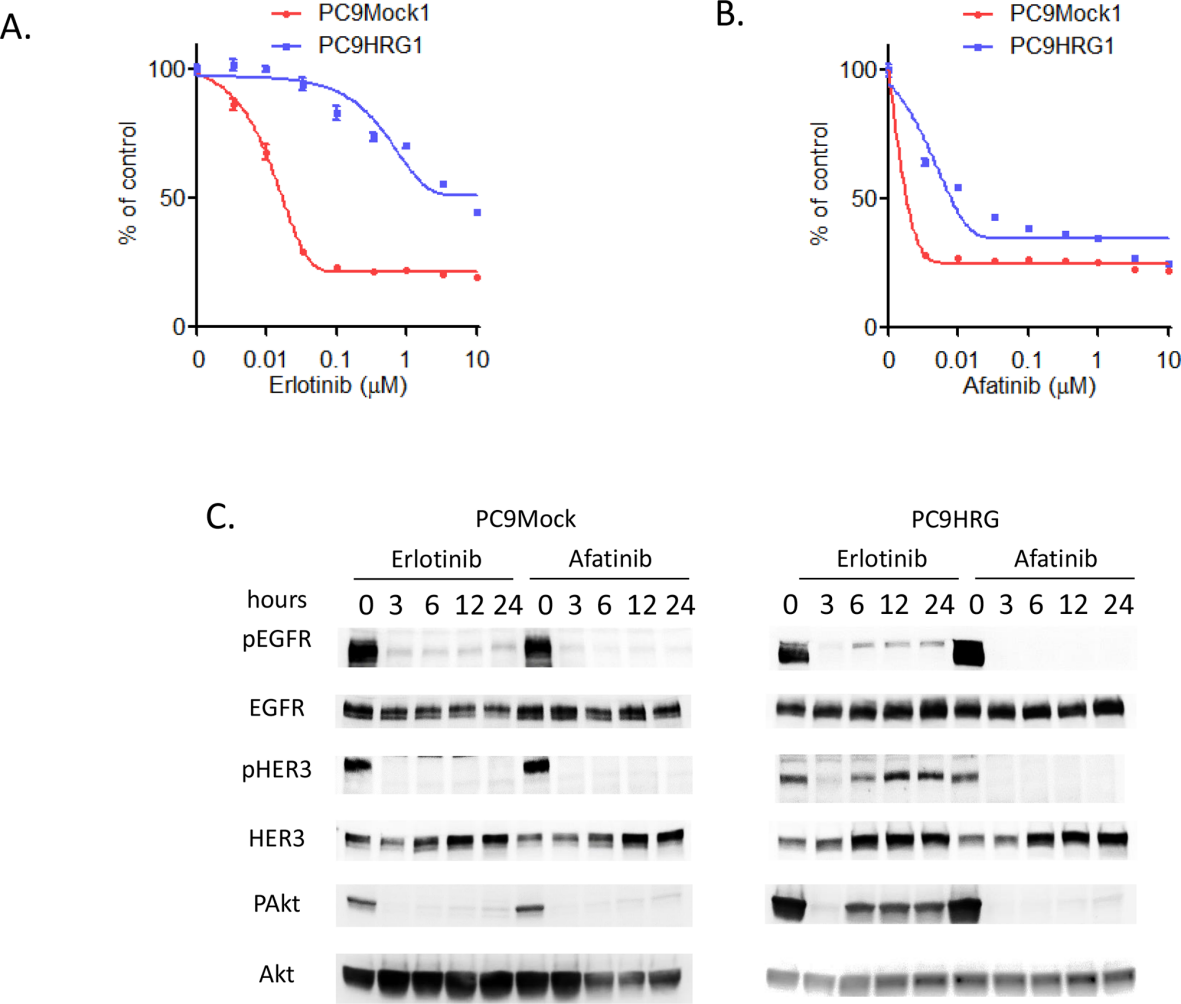
Heregulin-overexpressing NSCLC cell line PC9HRG cells are resistant to erlotinib, but sensitive to afatinib **A.** Mock-transfected EGFR mutant NSCLC PC9Mock cells or stably heregulin-transfected PC9HRG cells were treated with the indicated concentrations of erlotinib; cell viability was measured 3 days later and values are plotted relative to untreated control cells (means ± SD). **B.** PC9Mock cells or PC9HRG cells were treated with the indicated concentrations of afatinib; cell viability was measured 3 days later. **C.** PC9Mock and PC9HRG cells were treated with 1 μM erlotinib or 0.1 μM afatinib for the indicated times, then probed for the indicated proteins.

First, we evaluated the efficacy of erlotinib in PC9Mock and heregulin-overexpressing PC9HRG cells using an *in vitro* cell-proliferation inhibition assay. Both cell lines were treated with erlotinib in doses ranging from 0.0033 to 10 μM for 72 h. Similar to our previous study, PC9Mock cells showed decreased numbers of viable cells after erlotinib treatment in a dose-dependent manner, whereas PC9HRG cells maintained cell-proliferation at higher concentration of erlotinib (Figure 1A) [22]. Next, we evaluated the susceptibility to afatinib in these cell lines. Whereas the PC9HRG cells were refractory to erlotinib, they remained sensitive to afatinib (Figure 1B). Thus, the IC_50_ (the concentration required to effect 50% cell growth inhibition) value of erlotinib in PC9HRG cells was approximately 5 μM, whereas the IC_50_ value of afatinib was approximately 20 nM. According to the pharmacokinetic data for afatinib, the mean steady-state maximum plasma concentration (Cmax) of afatinib at the FDA-approved dosing (40 mg/day) is 78 nM [27]. Thus, the IC_50_ value of afatinib in PC9HRG cells was much less than the clinically achievable plasma concentration of afatinib in patients with NSCLC. We also evaluated another second generation EGFR-TKI, dacomitinib, for inhibitory ability against PC9HRG cell proliferation. PC9HRG cells were sensitive to dacomitinib as well, with an the IC_50_ value of approximately 10 nM (Supplementary Figure S1).

Here, we hypothesized that the differential sensitivity between erlotinib and afatinib in heregulin overexpressing PC9HRG cells was the result of differing signaling transduction, especially in the HER3-AKT signaling pathway, as our previous study had shown that refractoriness to erlotinib is caused by HER3 re-activation in PC9HRG cells [22]. We therefore evaluated this cell signaling pathway in PC9Mock and PC9HRG cells, which were treated with erlotinib or afatinib for 24 h, using immunoblotting (Figure 1C). This analysis demonstrated that the phosphorylation of EGFR as well as HER3 was decreased in PC9Mock cells following either erlotinib or afatinib exposure. Furthermore, both drugs decreased the phosphorylation of AKT, a downstream effector of HER3, in PC9Mock cells. The phosphorylation of EGFR was also decreased in heregulin-overexpressing PC9HRG cells following erlotinib exposure. However, the phosphorylation of HER3 was decreased in PC9HRG cells following 3 h erlotinib exposure, but HER3 was re-activated after 6 h erlotinib exposure, which was accompanied by increased total HER3 expression. In these cells, AKT was also reactivated after 6 h erlotinib exposure. These observations were identical to those from our previous study. However, in contrast to the results following erlotinib treatment, afatinib maintained the inhibition of both EGFR and HER3 phosphorylation in heregulin-overexpressing PC9HRG cells during 24 h despite increased total HER3 levels. Finally, afatinib exposure maintained the inhibition of phosphorylation of AKT in these cells over 24 h. These results suggested that the different susceptibilities to erlotinib and afatinib are caused by different capabilities of those drugs to inhibit the HER3-AKT signaling pathway in heregulin overexpressing PC9HRG cells.

### Afatinib can inhibit HER3 activation accompanied with pan-HER family inhibition in heregulin-overexpressing PC9HRG cells

**Figure 2:**
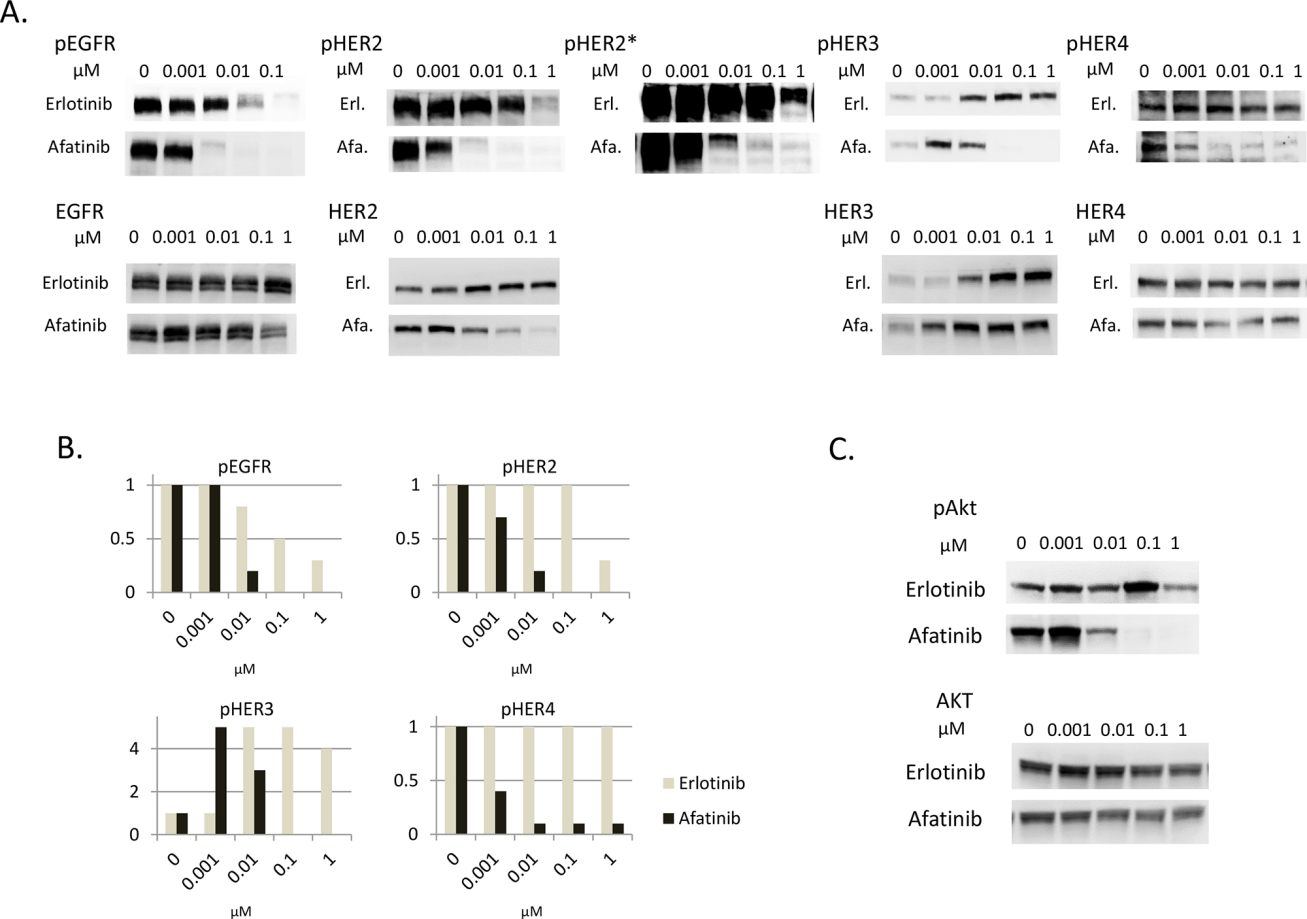
Afatinib inhibits phosphorylation of HER3 dose-dependently, in parallel with inhibition of phosphorylation of other HER family members in heregulin-overexpressing NSCLCs **A, B.** PC9HRG cells were treated with the various indicated concentrations of erlotinib or afatinib for 12 h, then probed for the indicated proteins (A) Phosphor-HER family protein expression levels were measured and quantified using the Multi Gauge system (Fujifilm, Japan) (B) X axis; concentration of drugs, Y axis; fold changes compared with untreated control. *; with long exposure. **C.** PC9HRG cells were treated with the various indicated concentration of erlotinib or afatinib for 12 h, then probed for the indicated proteins.

The susceptibility to afatinib seems to be correlated with the inhibition of HER3-AKT signaling in PC9HRG cells, although HER3 has weak kinase activity that is not sufficient for auto-phosphorylation. Others have reported that HER3 can be trans-phosphorylated by other HER family receptors in cancer tissues [28, 29]. In addition, our previous study observed that HER3 associated with HER2 in PC9HRG cells treated with erlotinib [22]. Therefore, we expected that afatinib inhibited the tyrosine kinase activities of other HER family receptors and thus prevented the trans-phosphorylation of HER3 in PC9HRG cells. To test this hypothesis, we assessed the total and phosphorylated levels of HER family proteins including EGFR, HER2, HER3, and HER4 in heregulin-overexpressing PC9HRG cells treated with erlotinib or afatinib. PC9HRG cells were treated with erlotinib or afatinib at doses ranging from 0.001 to 1 μM for 12 h. Phosphorylation of EGFR was found to be decreased in PC9HRG cells treated with erlotinib as well as afatinib in a dose-dependent manner (Figure 2A, 2B).

We also noted that phosphorylation of HER2 was not inhibited by exposure to erlotinib at concentrations of 0.1 μM or less. Furthermore, the results from immunoblots with long exposures showed that HER2 phosphorylation still remained even following 1 μM erlotinib exposure. In contrast, phosphorylation of HER2 was drastically inhibited with afatinib at 0.01 μM. In addition, total HER2 levels were also decreased in cells treated with afatinib in a dose-dependent manner, although total HER2 levels were maintained in cells treated with erlotinib.

Third, we observed that phosphorylation of HER3 was increased in cells treated with 0.01 μM or higher concentrations of erlotinib accompanied with elevated total HER3 expression levels. Phosphorylation of HER3 was also increased in cells treated with 0.001 μM afatinib; however, this was decreased in cells treated with 0.01 μM or higher concentrations of afatinib, even though total HER3 expression levels were increased.

Finally, phosphorylation of HER4 was not shown to be decreased in cells treated with erlotinib at even 1 μM concentration. In contrast to erlotinib exposure, phosphorylation of HER4 was inhibited in cells treated with afatinib in a dose-dependent manner.

Therefore, these results showed that afatinib could inhibit phosphorylation of all HER family proteins in a dose-dependent manner; the phosphorylation of HER3 was especially well paralleled with that of other HER family members including EGFR, HER2, and HER4 in PC9HRG cells. These results suggest that afatinib prevents trans-phosphorylation of HER3 by inactivating other HER family kinases. In addition, phosphorylation of AKT, the downstream effector of HER3, was potently inhibited by afatinib but not by erlotinib in a dose-dependent manner (Figure 2C). This suggests that the HER3-AKT signaling pathway was inhibited by afatinib, but not by erlotinib.

### Afatinib, but not erlotinib, can induce apoptosis in heregulin-overexpressing PC9HRG cells

**Figure 3:**
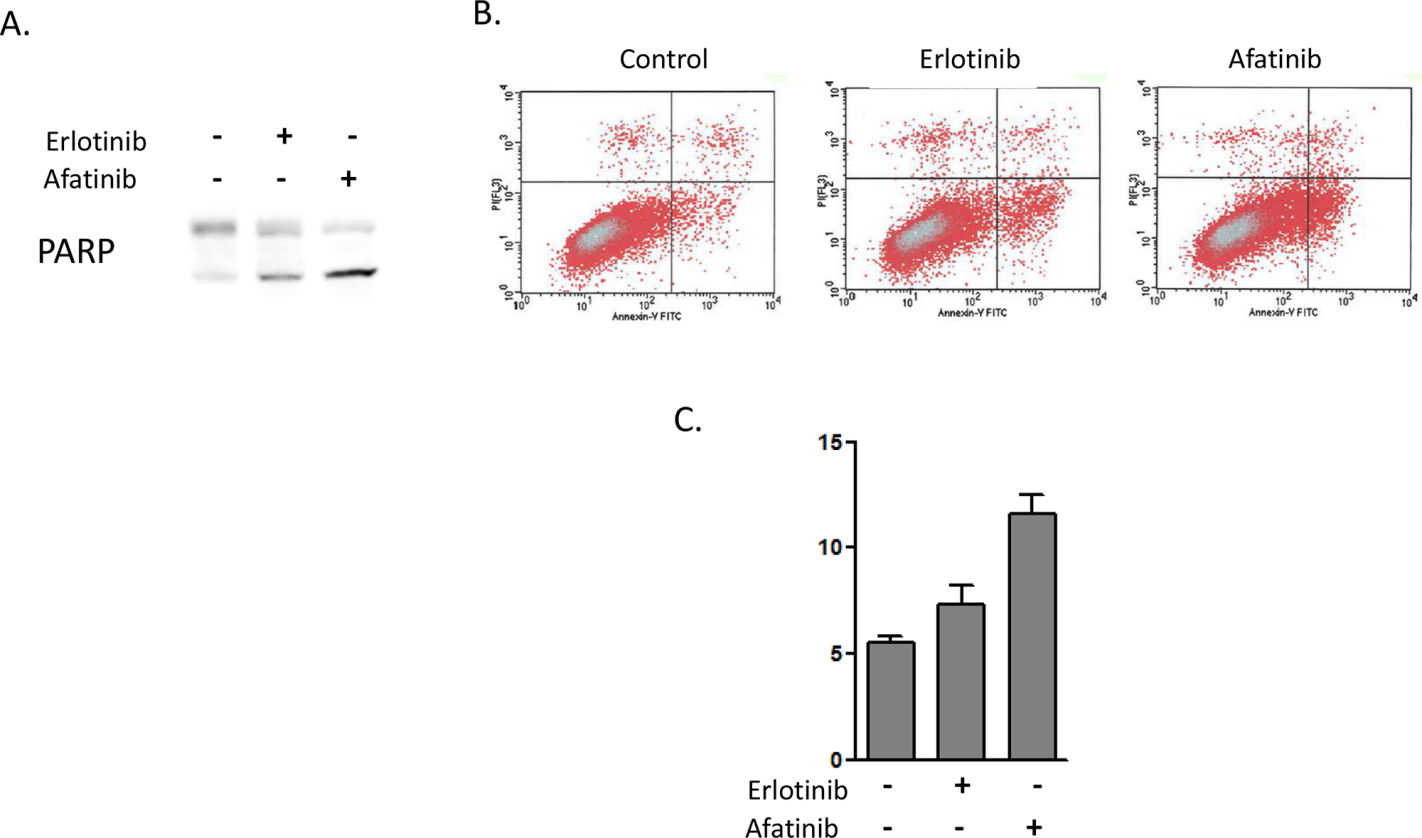
Afatinib induces higher levels of apoptosis in heregulin-overexpressing NSCLCs than does erlotinib **A.** PC9HRG cells were treated with 1 μM erlotinib or 0.1 μM afatinib for 24 h, then were probed for the indicated protein. **B, C.** PC9HRG cells were treated with 1 μM erlotinib or 0.1 μM afatinib for 6 h, then the number of apoptotic cells was determined by staining with propidium iodide (PI) and fluorescein isothiocyanate (FITC)-labeled annexin V followed by flow cytometry. Representative flow cytometric profiles are shown in (B), and quantitative data (means ± SE of three independent experiments) are shown in (C).

We observed that afatinib, but not erlotinib, could inhibit HER3-AKT signal transduction in heregulin-overexpressing PC9HRG cells. This pathway generally plays a key role in anti-apoptosi; therefore, we examined whether afatinib could induce apoptosis in heregulin-overexpressing PC9HRG cells. PC9HRG cells were treated with 1 μM erlotinib or 0.1 μM afatinib for 24 h, and then poly (ADP ribose) polymerase (PARP) cleavage was evaluated using immunoblotting. Afatinib caused more PARP cleavage compared to erlotinib in PC9HRG cells (Figure 3A). We also evaluated apoptosis using an annexin V binding assay. Consistent with PARP cleavage results, afatinib exposure significantly increased the proportion of apoptosis in PC9HRG cells compared with that obtained following erlotinib exposure (Control vs erlotinib vs afatinib; 5.5 vs 7.4 vs 11.6%, respectively; *P* < 0.05, Figure 3B, 3C). These results suggest that PC9HRG cells are more sensitive to afatinib than to erlotinib because HER3-AKT signaling inhibition by afatinib causes a predisposition to apoptosis in these cells.

### Heregulin-overexpressing PC9HRG xenografts showed sensitivity to afatinib but not erlotinib

**Figure 4:**
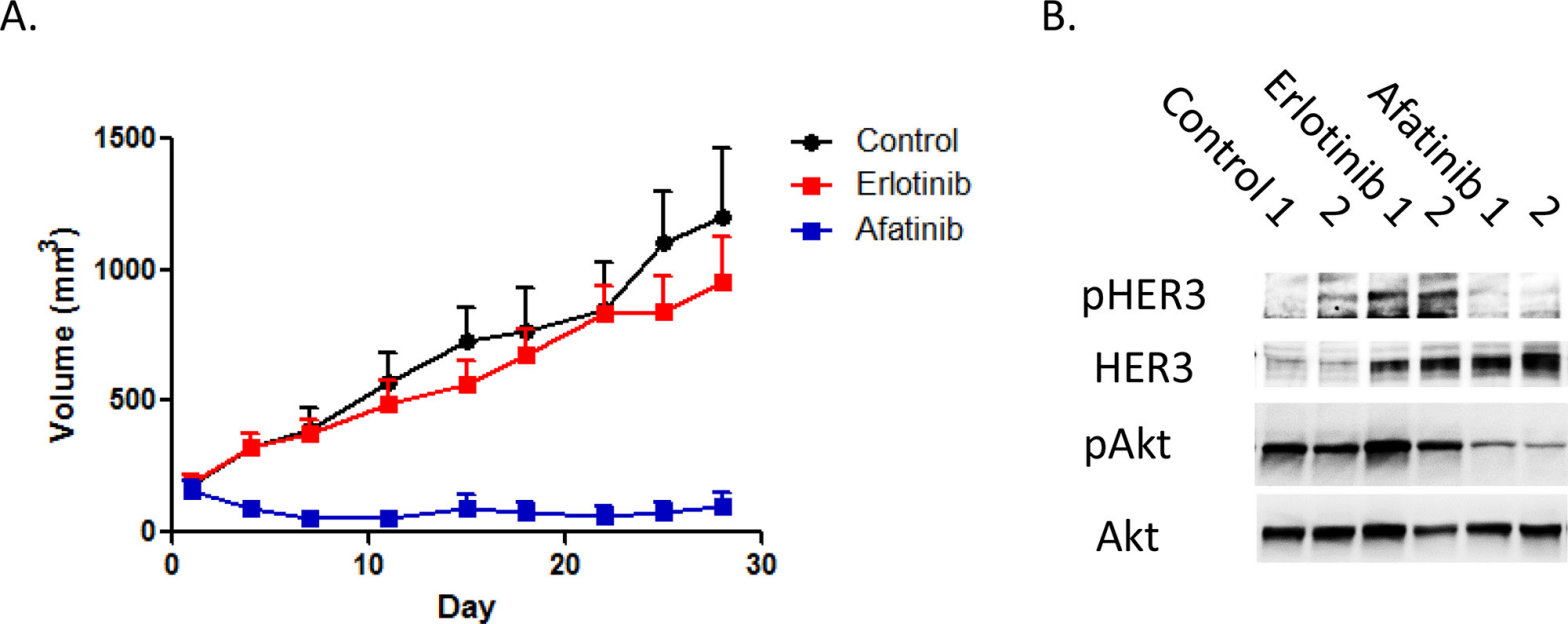
Heregulin-overexpressing NSCLC cell xenografts are resistant to erlotinib, but sensitive to afatinib **A.** In a xenograft NSCLC model, heregulin-overexpressing PC9HRG cells were injected subcutaneously into nude mice, and tumors were allowed to grow to at least 150 mm^3^ before animals were treated with erlotinib, afatinib, or the vehicle. Each treatment group consisted of *n* = 10 mice. Data represent means ± SEM. *P < 0.05. **B.** PC9HRG cell xenografted mice were treated with erlotinib, afatinib, or vehicle for 12 h; cell lysates from tumors were probed for the indicated proteins.

To verify whether these *in vitro* findings were relevant *in vivo*, heregulin-overexpressing PC9HRG cells were implanted subcutaneously in mice, which were then treated with erlotinib, afatinib, or vehicle for four weeks. Xenograft tumors arising from PC9HRG cells continued to grow with erlotinib treatment, while tumors shrank with afatinib treatment (Figure 4A). We also evaluated the HER3-AKT signaling pathway in tumors using immunoblotting. Consistent with the results from the *in vitro* study, erlotinib treatment increased phosphorylation of HER3 accompanied with increased total HER3 levels (Figure 4B). In addition, we also noted that erlotinib could not inhibit AKT phosphorylation in the tumors. In contrast, afatinib treatment evinced decreased phosphorylation of HER3 even though the total HER3 levels increased (Figure 4B). Finally, afatinib was able to inhibit the phosphorylation of AKT in tumors. Together, these results suggested that afatinib treatment *in vivo* was also able to overcome heregulin-induced EGFR-TKI resistance through alteration of HER3-AKT signal transduction.

### A subpopulation of patients with EGFR mutation and NSCLC has high plasma concentrations of heregulin and might be sensitive to afatinib even after acquisition of erlotinib-resistance

**Figure 5:**
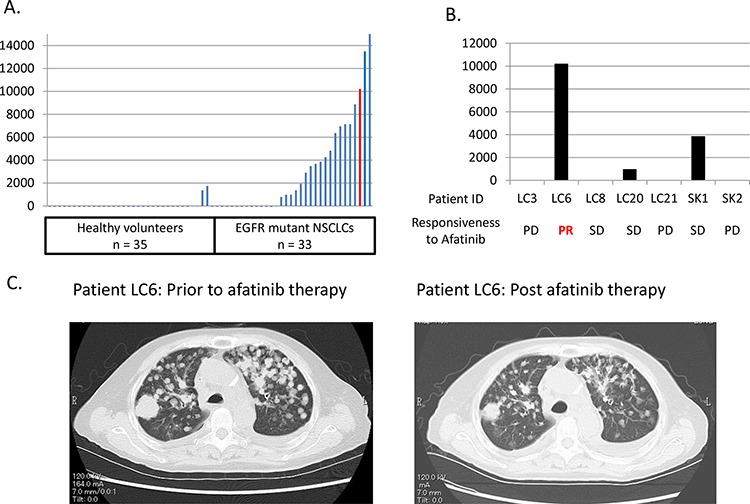
A patient with an EGFR mutation and NSCLC, with high plasma heregulin shows resistance to erlotinib, but is sensitive to afatinib **A.** Plasma heregulin expression levels were measured using ELISA in healthy volunteers (*n* = 35) and in patients with EGFR mutations and NSCLC (*n* = 33). The red bar indicates the plasma heregulin expression level of a patient, LC6, who acquired resistance to erlotinib and then achieved a response with afatinib therapy. **B.** Seven patients were treated with afatinib after acquiring resistance to first generation EGFR-TKIs including gefitinib or erlotinib. Plasma heregulin expression levels were measured in those patients post-acquisition of resistance to first generation EGFR-TKIs. The responsiveness to afatinib therapy is shown for each patient. **C.** Chest CT scan of a patient, LC6, is shown prior to and post afatinib therapy.

To verify whether these preclinical findings were relevant in a clinical situation, we evaluated plasma heregulin expression levels among healthy individuals and patients with EGFR mutations and NSCLC, and assessed their responsiveness to afatinib. Among healthy volunteers (*n* = 35) plasma heregulin levels were minimal (median 0 pg/mL; 0–1750 pg/mL, Figure 5A). However, patients with EGFR mutations and NSCLC (*n* = 33) had variable and significantly higher levels of plasma heregulin than did the healthy volunteers (median 981 pg/mL; 0–16,181 pg/mL). Among the patients, seven were treated with afatinib after failure to respond to gefitinib or erlotinib treatment owing to tumor progression. Those patients were found to be also refractory to afatinib except for one, LC6, who achieved partial response with afatinib therapy (Figure 5B, 5C, Supplementary table S1). Plasma heregulin levels were measured prior to afatinib treatment; among these, LC6 exhibited a relatively high level of plasma heregulin.

## DISCUSSION

A novel treatment strategy for overwhelming EGFR-TKI resistance is required for improving the prognosis for patients with NSCLC harboring EGFR activating mutations. Our previous study showed that heregulin overexpression caused erlotinib resistance in EGFR mutant NSCLC cells [22]. Here we showed that in pre-clinical studies, afatinib can overcome the resistance to first generation EGFR-TKIs such as erlotinib in patients with heregulin-overexpressing NSCLCs. Mechanistically, in contrast to erlotinib, afatinib could inhibit the phosphorylation of pan-HER family member proteins at clinically achievable concentrations in heregulin-overexpressing PC9HRG cells. Afatinib has previously been shown to directly inhibit the kinase activity of EGFR, HER2, and HER4 [10]. HER3, in contrast, has only weak kinase activity, but is a potent activator of its binding partner, HER2 [28, 29]. Our previous study had shown that HER3 preferentially heterodimerized with HER2, which caused an increase in the phosphorylation of HER3 in PC9HRG cells after erlotinib treatment [22]. In addition, we demonstrated that the anti-HER2 antibody pertuzumab in combination with erlotinib decreased the phosphorylation of HER3 and its downstream effector AKT in those cells [22]. Together, these results suggested that afatinib inhibited members of the pan-HER family, especially EGFR, as well as inhibiting HER2 kinase activity, which reduced the trans-phosphorylation of HER3 in PC9HRG cells.

In a clinical study, we observed here that a patient with NSCLC carrying an EGFR mutation and exhibiting high plasma heregulin expression achieved a response with afatinib treatment, despite having previously developed erlotinib resistance. Comparatively, our previous analysis using archival tissue samples showed that a subpopulation of NSCLC tumors expressed elevated levels of heregulin following development of gefitinib resistance, versus those observed prior to EGFR-TKI therapy [22]. Here, we were not able to evaluate whether plasma and tumor heregulin expression levels were correlated. However, the higher plasma heregulin levels observed in patients with NSCLC compared to healthy volunteers suggested that plasma heregulin might be derived from the tumors themselves. Furthermore, previous clinical trials have shown that a subpopulation of patients with EGFR mutations and NSCLC remained sensitive to subsequent afatinib therapy, although they had previously acquired resistance to gefitinib or erlotinib treatment [16, 17]. Thus, these results suggested that a subpopulation of patients with EGFR mutations and NSCLC developed first generation EGFR-TKI resistance because of increased heregulin expression, but that such patients might retain afatinibsensitivity.

In addition, afatinib might be the optimum treatment choice for EGFR-TKI naïve patients with NSCLC and high heregulin expression. Previously, heregulin-overexpression was correlated with the shortness of progression-free survival in patients with NSCLC treated with erlotinib [25, 26]. However, our previous preclinical study showed that use of the anti-HER3 antibody patritumab in combination with erlotinib was able to overcome the heregulin-induced EGFR-TKI resistance in PC9HRG cells [22]. Furthermore, patritumab in combination with erlotinib also significantly prolonged progression-free survival compared with placebo in combination with erlotinib in a subpopulation of patients with NSCLC and high expression levels of heregulin in a randomized clinical trial [25]. Similarly, our current study suggests that afatinib might be more optimized than first generation EGFR-TKIs for use in EGFR-TKI naïve patients with NSCLC and high heregulin expression. Additionally, it has recently been shown that the survival benefits of afatinib treatment are greater than those obtained with erlotinib in patients with EGFR “wild-type” NSCLC [30]. However, no predictive biomarker was identified for the outcome of afatinib therapy among these patients, although some patients with EGFR wild-type NSCLC were shown to exhibit high levels of heregulin expression in other studies [25, 26]. Thus, heregulin expression analysis might be helpful to enhance the benefit of afatinib use in patients with EGFR wild-type NSCLC as well.

In summary, this study is the first demonstration of the efficacy of a second generation EGFR-TKI, afatinib, against NSCLC refractory to first generation EGFR-TKIs because of overexpression of a kinase ligand, heregulin. Our results, combined with those from the published literature, suggest that the unique property of afatinib as a HER-family TKI underlies its efficacy therein. Therefore, we recommend that afatinib should be examined in a clinical setting on a cohort of patients with NSCLC with first generation EGFR-TKI refractoriness and high heregulin expression.

## MATERIALS AND METHODS

### Cell culture and reagents

PC9Mock and PC9HRG cell lines have been previously characterized; these possess EGFR exon 19 deletions [22]. PC9HRGs were stably transfected with a full-length cDNA fragment encoding human heregulin (*NRG1*, NM_13956) as previously described [22]. Cells were cultured in Roswell Park Memorial Institute (RPMI)-1640 medium (Sigma-Aldrich, St. Louis, MO, USA) supplemented with 10% fetal bovine serum (FBS) and penicillin-streptomycin-amphotericin B (Wako Pure Chemical Industries, Ltd., Osaka, Japan). We used cell lines within 6 months from resuscitation. Afatinib and erlotinib were obtained from commercial sources. Stock solutions of 10 mM afatinib and erlotinib were prepared in dimethylsulfoxide and stored at −20°C.

### Antibodies and western blotting

Cells were seeded at a density of 1 × 10^6^ cells/plate in Prime Surface 60-mm plates (Sumitomo Bakelite Co. Ltd., Tokyo, Japan) and allowed to grow overnight in medium containing 0.5% FBS before the addition of the drug to the medium. Cells were incubated for various times with various concentrations of drugs, then washed with phosphate-buffered saline (PBS) and lysed in buffer containing 25 mM Tris (pH 8.3), 192 mM glycine, 0.1% sodium dodecyl sulfate, and 1 mM phenylmethylsulfonyl fluoride as previously described [2]. Cell lysates were centrifuged at 15,000 × *g* for 10 min at 4°C, and the supernatant was collected for subsequent procedures. Western blotting was performed following a standard protocol; samples were resolved by sodium dodecyl sulfate polyacrylamide gel electrophoresis and transferred to nitrocellulose membranes, which were probed with antibodies against phospho-AKT (Ser-473), AKT, phospho-EGFR (Tyr-1068), EGFR, phospho-HER3 (Tyr-1289), phospho-HER4, HER4, ERK, PARP, and heregulin (all from Cell Signaling Technology, Danvers, MA, USA); phospho-ERK1/2 (Thr-202/204) (Santa Cruz Biotechnology, Santa Cruz, CA, USA); β-actin (Sigma-Aldrich); and phospho-HER2 (Tyr-1248) and HER2 (Merck/Millipore, Darmstadt, Germany). Antibodies were used at dilutions recommended by the respective manufacturers, and signals were detected using an enhanced chemiluminescence system (GE Healthcare, Pittsburgh, PA, USA).

### Cell-proliferation inhibition assays

The sensitivity to afatinib and erlotinib was evaluated using the CellTiter-Glo Luminescent Cell Viability Assay (Promega, Madison, WI, USA). Briefly, cells were resuspended in medium containing 0.5% FBS in a Prime Surface 96U 96-well plate (Sumitomo Bakelite Co. Ltd., Tokyo, Japan) at 5 × 10^3^ cells/well. After overnight incubation, agents were added to the medium at 0.0033–10 μM for afatinib and erlotinib. Cell viability was quantified based on luminescence after the addition of the CellTiter-Glo reagent. All experimental points were set up in 6 wells.

### Annexin V binding assay

The binding of annexin V to cells was measured with the use of an Annexin-V-FLUOS Staining Kit (Roche diagnostics DmbH, Mannheim, Germany). Cells were harvested by exposure to trypsin-EDTA, washed with PBS, and centrifuged at 200 × g for 5 min. The cell pellets were resuspended in 100 μL Annexin-V-FLUOS labeling solution, incubated for 10 to 15 min at 15°C to 25°C, and then analyzed for fluorescence with a flow cytometer (FACSCalibur) and Cell Quest software (Becton Dickinson, Franklin Lakes, NJ, USA).

### Xenograft studies

Xenograft studies were performed using PC9HRG cells as previously described [22]. Briefly, cells were implanted into female athymic nude mice (BALB/cAJcl-nu/nu) and tumors were allowed to grow to at least 150 mm^3^ before mice were treated with afatinib (10 mg/kg) or erlotinib (25 mg/kg) administered daily by oral gavage. Each treatment group consisted of eight mice. Studies were performed in accordance with the standards of the Institutional Animal Care and Use Committee under a protocol approved by the Animal Care and Use Committee at Kinki University.

### Patients and treatment

The study included 33 patients who were treated for EGFR mutant NSCLC at Kinki University School of Medicine between September 2013 and March 2015. Most patients had received first generation EGFR-TKIs such as gefitinib or erlotinib. Serum of healthy volunteers was obtained from commercial sources. The study was approved by the Institutional Review Board of Kinki University School of Medicine. Written informed consent was obtained from all patients.

### Measurement of heregulin plasma concentrations

Plasma samples were obtained from 35 healthy volunteers and 33 patients with EGFR mutations and NSCLC. Plasma concentrations of heregulin were measured using commercially available enzyme-linked immunosorbent assay (ELISA) kits (NRG1 beta 1 human ELISA Kit, Abcam, Cambridge, MA, USA) according to the manufacturer's instructions. Briefly, samples and standards were added into a 96-well microplate coated with the capture antibody. The plates were washed, and the detection antibody was added. After addition of the chromogen, the color intensity was measured at 450 nm using a spectrophotometric plate reader. Heregulin protein concentrations were determined by comparison to the standard curve.

### Statistical analyses

Statistical analyses were performed using StatView v.5.01 software (SAS Institute, Cary, NC, USA). All statistical tests were two-sided, and a *P* value < 0.05 was considered statistically significant. Data were graphically displayed using GraphPad Prism v.5.0 for Windows (GraphPad Software, Inc., La Jolla, CA, USA).

## SUPPLEMENTARY FIGURE AND TABLE



## References

[1] Hynes NE, Lane HA (2005). ERBB receptors and cancer: the complexity of targeted inhibitors. Nat Rev Cancer.

[2] Yonesaka K, Zejnullahu K, Lindeman N, Homes AJ, Jackman DM, Zhao F, Rogers AM, Johnson BE, Jänne PA (2008). Autocrine production of amphiregulin predicts sensitivity to both gefitinib and cetuximab in EGFR wild-type cancers. Clin Cancer Res.

[3] Paez JG, Jänne PA, Lee JC, Tracy S, Greulich H, Gabriel S, Herman P, Kaye FJ, Lindeman N, Boggon TJ, Naoki K, Sasaki H (2004). EGFR mutations in lung cancer: correlation with clinical response to gefitinib therapy. Science.

[4] Lynch TJ, Bell DW, Sordella R, Gurubhagavatula S, Okimoto RA, Brannigan BW, Harris PL, Haserlat SM, Supko JG, Haluska FG, Louis DN, Christiani DC (2004). Activating mutations in the epidermal growth factor receptor underlying responsiveness of non-small-cell lung cancer to gefitinib. N Engl J Med.

[5] Sequist LV, Waltman BA, Dias-Santagata D, Digumarthy S, Turke AB, Fidias P, Bergethon K, Shaw AT, Gettinger S, Cosper AK, Akhavanfard S, Heist RS (2011). Genotypic and histological evolution of lung cancers acquiring resistance to EGFR inhibitors. Sci Transl Med.

[6] Kobayashi S, Boggon T, Dayaram T, Jänne PA, Kocher O, Meyerson M, Johnson BE, Eck MJ, Tenen DG, Halmos B (2005). EGFR mutation and resistance of non-small cell lung cancer to gefitinib. N Engl J Med.

[7] Engelman JA, Zejnullahu K, Mitsudomi T, Song Y, Hyland C, Park JO, Lindeman N, Gale CM, Zhao X, Christensen J, Kosaka T, Holmes AJ (2007). MET amplification leads to gefitinib resistance in lung cancer by activating ERBB3 signaling. Science.

[8] Takezawa K, Pirazzoli V, Arcila ME, Nebhan CA, Song X, de Stanchina E, Ohashi K, Janjigian YY, Spitzler PJ, Melnick MA, Riely GJ, Kris MG (2012). HER2 amplification: a potential mechanism of acquired resistance to EGFR inhibition in EGFR-mutant lung cancers that lack the second-site EGFRT790M mutation. Cancer Discov.

[9] Yano S, Wang W, Li Q, Matsumoto K, Sakurama H, Nakamura T, Ogino H, Kakiuchi S, Hanibuchi M, Nishioka Y, Uehara H, Mitsudomi T (2008). Hepatocyte growth factor induces gefitinib resistance of lung adenocarcinoma with epidermal growth factor receptor-activating mutations. Cancer Res.

[10] Li D, Ambrogio L, Shimamura T, Kubo S, Takahashi M, Chirieac LR, Padera RF, Shapiro GI, Baum A, Himmelsbach F, Rettig WJ, Meyerson M (2008). BIBW2992, an irreversible EGFR/HER2 inhibitor highly effective in preclinical lung cancer models. Oncogene.

[11] Sequist LV, Yang JCH, Yamamoto N, O'Byrne K, Hirsh V, Mok T, Geater SL, Orlov S, Tsai CM, Boyer M, Su WC, Bennouna J (2013). Phase III study of afatinib or cisplatin plus pemetrexed in patients with metastatic lung adenocarcinoma with EGFR mutations. J Clin Oncol.

[12] Wu YL, Zhou C, Hu CP, Feng J, Lu S, Huang Y, Li W, Hou M, Shi JH, Lee KY, Xu CR, Massey D (2014). Afatinib versus cisplatin plus gemcitabine for first-line treatment of Asian patients with advanced non-small-cell lung cancer harbouring EGFR mutations (LUX-Lung 6): an open-label, randomised phase 3 trial. Lancet Oncol.

[13] Yang JC, Wu YL, Schuler M, Sebastian M, Popat S, Yamamoto N, Zhou C, Hu CP, O'Byrne K, Feng J, Lu S, Huang Y (2015). Afatinib versus cisplatin-based chemotherapy for EGFR mutation-positive lung adenocarcinoma (LUX-Lung 3 and LUX-Lung 6): analysis of overall survival data from two randomised, phase 3 trials. Lancet Oncol.

[14] Engelman JA, Zejnullahu K, Gale CM, Lifshits E, Gonzales AJ, Shimamura T, Zhao F, Vincent PW, Naumov GN, Bradner JE, Althaus IW, Gandhi L (2007). PF00299804, an irreversible pan-ERBB inhibitor, is effective in lung cancer models with EGFR and ERBB2 mutations that are resistant to gefitinib. Cancer Res.

[15] Ercan D, Zejnullahu K, Yonesaka K, Xiao Y, Capelletti M, Rogers A, Lifshits E, Brown A, Lee C, Christensen JG, Kwiatkowski DJ, Engelman JA (2010). Amplification of EGFR T790M causes resistance to an irreversible EGFR inhibitor. Oncogene.

[16] Katakami N, Atagi S, Goto K, Hida T, Horai T, Inoue A, Ichinose Y, Koboyashi K, Takeda K, Kiura K, Nishio K, Seki Y (2013). LUX-Lung 4: A Phase II trial of afatinib in patients with advanced non-small-cell lung cancer who progressed during prior treatment with erlotinib, geﬁtinib, or both. J Clin Oncol.

[17] Miller VA, Hirsh V, Cadranel J, Chen YM, Park K, Kim SW, Zhou C, Su WC, Wang M, Sun Y, Heo DS, Crino L (2012). Afatinib versus placebo for patients with advanced, metastatic non-small-cell lung cancer after failure of erlotinib, gefitinib, or both, and one or two lines of chemotherapy (LUX-Lung 1): a phase 2b/3 randomised trial. Lancet Oncol.

[18] Jänne PA, Yang JC, Kim DW, Planchard D, Ohe Y, Ramalingam SS, Ahn MJ, Kim SW, Su WC, Horn L, Haggstrom D, Felip E (2015). AZD9291 in EGFR inhibitor-resistant non-small-cell lung cancer. N Engl J Med.

[19] Sequist LV, Soria JC, Goldman JW, Wakelee HA, Gadgeel SM, Varga A, Papadimitrakopoulou V, Solomon BJ, Oxnard GR, Dziadziuszko R, Aisner DL, Doebele RC (2015). Rociletinib in EGFR-mutated non-small-cell lung cancer. N Engl J Med.

[20] Zhou BB, Peyton M, He B, Liu C, Girard L, Caudler E, Lo Y, Baribaud F, Mikami I, Reguart N, Yang G, Li Y (2006). Targeting ADAM-mediated ligand cleavage to inhibit HER3 and EGFR pathways in non-small cell lung cancer. Cancer Cell.

[21] Aguilar Z, Akita RW, Finn RS, Ramos BL, Pegram MD, Kabbinavar FF, Pietras RJ, Pisacane P, Sliwkowski MX, Slamon DJ (1999). Biologic effects of heregulin/neu differentiation factor on normal and malignant human breast and ovarian epithelial cells. Oncogene.

[22] Yonesaka K, Hirotani K, Kawakami H, Takeda M, Kaneda H, Sakai K, Okamoto I, Nishio K, Jänne PA, Nakagawa K (2015). Anti-HER3 monoclonal antibody patritumab sensitizes refractory non-small cell lung cancer to the epidermal growth factor receptor inhibitor erlotinib. Oncogene.

[23] Yonesaka K, Zejnullahu K, Okamoto I, Satoh T, Cappuzzo F, Souglakos J, Ercan D, Rogers A, Roncalli M, Takeda M, Fujisaka Y, Philips J (2011). Activation of ERBB2 signaling causes resistance to the EGFR-directed therapeutic antibody cetuximab. Sci Transl Med.

[24] Schoeberl B, Faber AC, Li D, Liang MC, Crosby K, Onsum M, Burenkova O, Pace E, Walton Z, Nie L, Fulgham A, Song Y (2010). An ErbB3 antibody, MM-121, is active in cancers with ligand-dependent activation. Cancer Res.

[25] Mendella J, Freemana DJ, Fenga W, Hettmannb T, Schneiderb M, Blumb S, Ruhe J, Bange J, Nakamaru K, Chen S, Tsuchihashi Z, von Pawel J (2015). Clinical translation and validation of a predictive biomarker for patritumab, an anti-human epidermal growth factor receptor 3 (HER3) monoclonal antibody, in patients with advanced non-small cell lung cancer. EBioMedicine.

[26] Sequist LV, Lopez-Chavez A, Doebele RC, Gray JE, Harb WA, Modiano MR, Jackman DM, Baggstrom MQ, Atmaca A, Felip E, Provencio M, Cobo M (2014). A randomized phase 2 trial of MM-121, a fully human monoclonal antibody targeting ErbB3, in combination with erlotinib in EGFR wild-type NSCLC patients. J Clin Oncol.

[27] Wind S, Schmid M, Erhardt J, Goeldner RG, Stopfer P (2013). Pharmacokinetics of afatinib, a selective irreversible ErbB family blocker, in patients with advanced solid tumours. Clin Pharmacokinet.

[28] Zhang Q, Park E, Kani K, Landgraf R (2012). Functional isolation of activated and unilaterally phosphorylated heterodimers of ERBB2 and ERBB3 as scaffolds in ligand-dependent signaling. Proc Natl Acad Sci USA.

[29] Jura N, Shan Y, Cao X, Shaw DE, Kuriyan J (2009). Structural analysis of the catalytically inactive kinase domain of the human EGF receptor 3. Proc Natl Acad Sci USA.

[30] Soria JC, Felip E, Cobo M, Lu S, Syrigos K, Lee KH, Göker E, Georgoulias V, Li W, Isla D, Guclu SZ, Morabito A (2015). Afatinib versus erlotinib as second-line treatment of patients with advanced squamous cell carcinoma of the lung (LUX-Lung 8): an open-label randomised controlled phase 3 trial. Lancet Oncol.

