# Supplementary material for: The pan-HER family tyrosine kinase inhibitor afatinib overcomes HER3 ligand heregulin-mediated resistance to EGFR inhibitors in non-small cell lung cancer
Source: Oncotarget. 2015 Sep 15;6(32):33602–11. doi: 10.18632/oncotarget.5286 (PMC4741788; doi:10.18632/oncotarget.5286)
Supplement: Supplementary file 1 [file oncotarget-06-33602-s001.pdf]

SUPPLEMENTARY FIGURE AND TABLE

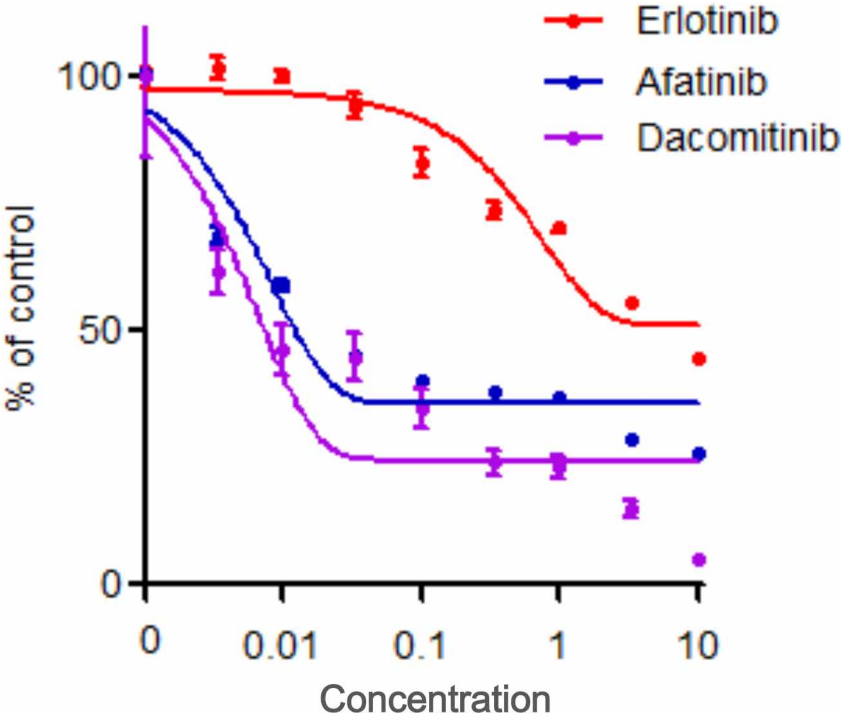

Supplementary Figure S1: Heregulin-overexpressing NSCLC cell line PC9HRG cells are resistant to erlotinib, but sensitive to afatinib, and dacomitinib. Stably heregulin-transfected PC9HRG cells were treated with the indicated concentrations of erlotinib, afatinib, and dacomitinib; cell viability was measured 3 days later and values are plotted relative to untreated control cells (means ± SD).

Supplementary Table S1: Previous treatments for Patient #LC6

Treatment number	Regimen	Treatment duration (mo)	Best Response
1st	CBDCA+PEM	7.0	SD
2nd	Gefitinib	9.4	SD
3rd	Docetaxel	18.0	SD
4th	Erlotinib+new drug	14.5	SD
5th	Erlotinib+new drug	2.9	PD
6th	Vinirelbine	3.5	SD
7th	S-1	1.6	PD
8th	Gemcitabine	2.0	PD
9th	3rd generation EGFR-TKI	2.8	PD

CBDCA, carboplatin; PEM, pemetrexed; EGFR-TKI, epidermal growth factor receptor tyrosine kinase inhibitor; mo, months; SD, stable disease; PD, progressive disease
